# Supervising Undergraduate Nursing Students on Their Bachelor's Thesis. A Qualitative Study

**DOI:** 10.1177/23779608231226074

**Published:** 2024-01-10

**Authors:** Guro Karlsholm, Beate André, Kjersti Grønning

**Affiliations:** 1 Department of Public Health and Nursing, 8018 Norwegian University of Science and Technology, Trondheim, Norway; 2 Department of Research, 60500 Nord-Trøndelag Hospital Trust, Levanger, Norway

**Keywords:** nursing student, nursing education, bachelor thesis, critical thinking, supervising

## Abstract

**Introduction:**

The bachelor's thesis (BT) in undergraduate education is often an autonomous and individual assignment. It aims to demonstrate knowledge gained through education, to be an assessment tool, and to give new learning outcomes through working on the thesis. The process of working with the BT most often includes individual supervision. There is an absence of support for teachers supervising undergraduate nursing theses, which includes a lack of national or institutional guidelines, role definitions and research material.

**Objective:**

Given the lack of helpful guidelines, this study aims to explore what the teachers emphasize as important regarding their supervision of nursing students writing their BT.

**Methods:**

The study has a qualitative approach, featuring individual semi-structured interviews with nursing teachers supervising nursing students on the BT. Reflexive thematic analysis is used as the method of analysis.

**Results:**

The analyses resulted in two generated themes, each with two subthemes, describing the patterns the teachers emphasize as important aspects of their supervision of nursing students writing their BT. The themes were “The pedagogical approach to the supervision” and “The advantages of the bachelor's thesis to the nursing profession.”

**Conclusions:**

The teachers balanced different roles in the supervision of the students. They wanted the students to grow through challenging them, grounded in a relationship of trust and confidence. They were role models for how to be critical thinkers and incorporated critical thinking as a pedagogical implement in supervision, wanting to raise the students to become nurses who think critically. The teachers sought to combine clinical relevance with academic literacy to secure a new generation of nurses able to be a part of the future development of the profession. They wanted to equip the students with knowledge, skills, and confidence to speak up and communicate nursing. Last, the teachers combined their clinical and academic identities in the supervision of the BT.

## Introduction

Most nursing education in Europe includes a final project at the end of the education (Humar & Sansoni, 2017). The project, or assignment, is named with diverse terminology—e.g., final-year dissertation or project, undergraduate dissertation or bachelor's thesis (Reguant et al., 2018). This article uses the term “bachelor's thesis” (BT). The BT is often designed as a small research project (Reguant et al., 2018) which can be informed by theory or empirical study. It is not regarded as a full-scale research project, but a platform to develop research skills, competencies and scientific curiosity at the undergraduate level (Gallart et al., 2015). Since most undergraduate students are inexperienced in research, they must develop these skills and competencies during their work on the BT (Agricola et al., 2018). They will need individual support during supervision, adapted to the student's level of knowledge and need for support (Agricola et al., 2021).

Supervision research projects are described as a complex and subtle form of teaching which includes both the teaching process for the supervisor and the learning process for the student (Agricola et al., 2021). Nevertheless, there is an absence of support for undergraduate thesis supervisors, which includes a lack of national or institutional guidelines, role definitions, training, research material, and theoretical frames of reference (Ädel et al., 2023; Holmberg, 2006; Roberts & Seaman, 2018; Shanahan et al., 2015; Todd et al., 2006).

## Review of Literature

### The Bachelor's Thesis

The BT in undergraduate education is often an autonomous and individual assignment (Reguant et al., 2018) that includes individual supervision (Gallart et al., 2015) and is the student's first big academic task. One of the aims of the BT is to let the students demonstrate the knowledge they have gained through their education, being summative and functioning as the culmination of the degree process (Feather et al., 2014; Roca et al., 2018). It also functions as an assessment tool for students’ competencies and knowledge gained through education (Gallart et al., 2015; Todd et al., 2004) and allows the students to address the actual learning outcomes and knowledge gained through working on the task. Essential learning outcomes, in addition to nursing knowledge, are to increase students’ academic literacy and research skills, to enhance their ability to write and develop critical thinking, and to enable them to become familiar with evidence-based practice, thus helping the students in their professional identity and academic growth (Reguant et al., 2018; Roberts & Seaman, 2018). The BT offers a disciplinary framework to understand and develop academic literacy and research skills related to their profession (Ashwin et al., 2017; Jefferies et al., 2018).

Through the BT, students learn to write but they also write to learn (Hunker et al., 2014). It is argued that writing, through formulating arguments, evaluating evidence, and connecting ideas, can support the development of critical thinking skills (Jefferies et al., 2018). Critical thinking is one of the characteristics of academic texts (Borglin & Fagerström, 2012); conversely, academic literacy increases critical thinking (Jefferies et al., 2018). Critical thinking is an essential part of the nursing profession (Borglin & Fagerström, 2012); it is discipline-specific and needs to be taught to undergraduate nursing students in a nursing context (Jefferies et al., 2018). Teachers play a vital role in students’ development of critical thinking: they need to model critical thinking for the students in all aspects of nursing education (Borglin & Fagerström, 2012). Teachers are encouraged to incorporate critical thinking into their teaching methods to increase students’ ability to learn critical thinking (Borglin & Fagerström, 2012; Jefferies et al., 2018). Such teaching must be tailored to each student's needs (Jefferies et al., 2018). The relationship between academic writing and critical thinking must be explicitly clarified or explained to the students (Borglin & Fagerström, 2012).

Newly graduated nurses have reported that working on the BT generated transferable knowledge in research and academic skills that they used in their daily work and as a method of learning (Lundgren & Halvarsson, 2009; Lundgren & Robertsson, 2013). They reported a more critical approach to the research process and increased critical thinking related to their work as nurses (Aguayo-González et al., 2020; Lundgren & Robertsson, 2013).

In cases where undergraduate nursing students have been part of small research projects which gave them real practical experience of the different steps in the research process, students reported increased competency in their use of tools to close the gap between theory and practice (de Jong et al., 2018), increased knowledge and positive attitude toward evidence-based practice (André et al., 2016), hands-on experience and motivation to initiate clinical projects in the future (Grønning et al., 2022), greater understanding of the application of theory in practice, and increased confidence in interacting with colleagues because of their new knowledge (Parkes & O'Dell, 2015).

### Supervising the BT

Due to the lack of unified guidelines, supervision is often based on individual preferences and competencies, with potential differences between the supervisors, and can become a stressful experience (Ädel et al., 2023; Holmberg, 2006; Roberts & Seaman, 2018). Some researchers have tried to identify common practices for supervising at the undergraduate level (Shanahan et al., 2015); however, more research is needed to be able to build a strong foundation for future recommendations for research supervision in nursing education.

Nevertheless, there is a repertoire of roles available for supervisors at the undergraduate level, not only based on individual experience but also dependent on culture and context. The roles range from being based on personal commitment (interactional) on one side to institutional roles (transactional) on the other (Ädel et al., 2023). The interactional side of the scale describes roles with a supportive approach, which takes the individual student as the starting point for supervision (Ädel et al., 2023). Interactional roles may use pep talks, show enthusiasm and empathy, and give a sense of security. They place the responsibility on the student but give support, help, and guidance along the way. They motivate students to learn and help them to understand how, what, and why. The teacher's focus is on the learning rather than the product. They also withhold intervention to support the independence and freedom of the student (Ädel et al., 2023).

Others describing supervisory roles at the interactional end of the scale describe a humane, approachable, and friendly teacher (Vehviläinen & Löfström, 2016), where the relationship between student and supervisor and the feedback from the teacher might have a positive impact on the student's academic outcomes (Jefferies et al., 2018). The supervision takes as its starting point social needs, such as emotions, expectations, and motivation (Agricola et al., 2021), making a comfortable and trusting relationship with a focus on confidence, support, and encouragement (Roberts & Seaman, 2018). The supervisor is described as a facilitator of the process, helping each student to attain the best possible result (Reguant et al., 2018) and fostering development, growth, and independence (Roberts & Seaman, 2018). A personal relationship between student and supervisor is a natural consequence of this type of role (Reguant et al., 2018; Roberts & Seaman, 2018). There is diversity among the students (Vehviläinen & Löfström, 2016), exemplified in their level of knowledge, personal abilities, expectations, and achievement (Reguant et al., 2018). This means that the supervisors must give the students personal attention, be sensitive to each individual student, and support them where they are in the process (Agricola et al., 2018).

On the transactional side of the scale, we find supervisory roles that are more institutional and more distanced from a personal relationship with the student (Ädel et al., 2023). These roles emphasize professional knowledge of the subject matter and provide information, instructions and corrections. They demand a minimum standard and high quality and may be seen as a reflection of the institutional surrounding of the thesis, the assessment and grading (Ädel et al., 2023).

Others have described transactional roles with supervisors helping the students to plan their time, set goals, and organize themselves and teaching them how to be self-critical (Reguant et al., 2018; Todd et al., 2006). There are descriptions of supervisors shepherding the process (Reguant et al., 2018), but also being aware not to push the students in any direction, since they must take responsibility for their own BT (Todd et al., 2006). Furthermore, it is the supervisor's responsibility to ensure fairness among the students through the fair and equal allocation of time for supervision (Vehviläinen & Löfström, 2016).

Supervision of the undergraduate dissertation is a negotiated practice between supervisor and student (Roberts & Seaman, 2018), where dialogue and communication are crucial since supervision is a two-way process (Agricola et al., 2018). The roles are not determined throughout the whole process but can change from being involved, directive and hands-on to being more in the background, acting on the student's initiative (Todd et al., 2006). The supervisor may shift between indirect and direct strategies (Agricola et al., 2021), and different roles can be taken when supervising the process or the product (Ädel et al., 2023). A difficult situation, though, is balancing being supportive and giving instruction and direction versus being challenging and encouraging the student's autonomy and independence (Agricola et al., 2018; Holmberg, 2006; Todd et al., 2006; Vehviläinen & Löfström, 2016). Balancing the roles is expressed as a problem or challenge in supervision, not a solution (Vehviläinen & Löfström, 2016). This confirms that the supervision process is complex, involving communication, collaboration, and the possibility of conflict (Agricola et al., 2018).

### Purpose of the Study

By letting the teachers speak about the supervision of the BT, we extend the understanding of being a supervisor of the BT in nursing education, which might contribute to future recommendations and guidelines for supervision in this specific field. We applied a broad approach to this study and asked the teachers about their thoughts on supervising the BT and about the learning outcomes from the BT, guided by the following research question: What do the teachers emphasize as important regarding their supervision of nursing students writing their BT?

## Methods

### Design

A qualitative approach was chosen for the study involving individual semi-structured interviews with teachers supervising the BT. The study is conducted within a constructivist approach, with a belief in knowledge as constructed in the interaction between the researcher, the participants, and the context (Braun & Clarke, 2022; Mann & MacLeod, 2015). Our hermeneutic orientation is such that the interpretations should stay close to the data, being recognizable to the participants. Reflexive thematic analysis is used as a method of analysis. The method is suitable to search for patterns of meanings across the dataset, allowing us to search for both semantic and latent meanings in the text (Braun & Clarke, 2022).

### Context

The location for this project is a nursing course at a university campus in Norway. The BT in this nursing institute is included in a course constituting 15 credits from the European Credit Transfer and Accumulation System. The course includes lectures on research methods, a course in literature search organized by a librarian, three group seminars with other students, and two mandatory individual supervisions, a third being voluntary. The BT is the last course the students fulfill within their 3-year nursing education resulting in a bachelor's degree. The students write their thesis individually and can choose between two types of paper: a literature review of approximately 10,000 words or participating in a clinical research project and writing an empirical paper as an academic article of approximately 4,500 words. The teachers are allocated as supervisors for the students based on the student's preliminary choice of theme or research question, presented in their compulsory process outline. The supervisor often has expert knowledge on the topic at hand, but not in all cases. The content of the course is described, together with predefined learning outcomes, in official documents available to teachers, students, and other interested parties on the university's internet homepage. The documents work as a guide for the teachers and a goal for the students. No other guidelines exist for the teachers on how to supervise the students.

### Recruitment and Data Collection

The study population was teachers supervising last year's undergraduate nursing students writing their BT. At the time of data collection, 28 teachers were supervising 192 students individually. To achieve purposive sampling for the quality and accuracy of the data collection, all teachers who had supervised the BT in the spring term of 2019 were invited to participate in the study. Invitations to participate were sent by email. Twelve teachers volunteered for the interviews by responding to the email from the first author. Time and place were decided individually. The leadership at the institute approved the interviews being conducted during the teachers’ working hours in an appropriate, but neutral, room at their workplace. The interviews were completed between June and August 2019, after the students had submitted their theses and graduated from education (June 2019). The duration of the interviews was 45 to 90 min, with an average of approximately 60 min. Data saturation was reached after the 12 interviews, no more interviews were needed.

A semi-structured interview guide was used with questions including learning outcomes from writing a BT, the construction of knowledge in nursing, and reflections on supervising the BT (Table 1). The first interview was listened to by all authors for discussion and acceptance of the questions. The review did not lead to any changes in the interview guide. The interviewees were encouraged to share their thoughts and use their own words and were given freedom and the possibility to speak freely. The researcher added questions for elaboration or clarification purposes and got the conversation back to the theme if needed. The first author conducted all interviews and transcribed them verbatim. In a few cases, repetition of words and special expressions were not transcribed for the readability of the excerpt and to maintain the anonymity of the informants.

**Table 1.** Examples from the Interview Guide.

Themes	Examples of questions
Learning outcomes from writing a BT	What would you say is the most important learning outcome of the bachelor's thesis? In what way do the students achieve the most important learning outcomes?
The construction of knowledge in nursing	Do you think the students have developed a different view of knowledge after writing a BT? How do you supervise the students so that they gain increased knowledge about EBP?
Reflections on supervising the BT	Do you supervise the product or the process? Awareness around different learning objectives? Are the students aware of the different learning outcomes linked to product and process? Does it require a different approach from the teacher to supervise a student in a literature review vs an empirical project?

*Note*. BT = bachelor’s thesis.

### Analysis

We carried out the analysis according to Braun and Clarke (2022) guidelines for reflexive thematic analysis and its six nonlinear phases. The interviews were transcribed and familiarized (phase 1) in the same order as they were conducted. In the coding phase (phase 2), the order was changed to break the possibility of a familiar flow in the dataset and to give a fresh perspective, minimizing the possibility of an unevenly coded dataset (Braun & Clarke, 2022). A preliminary research question guided a systematic inductive coding of the data material, coding only what was considered relevant to the research question. The first author did the coding alone, which is normal and good practice in reflexive thematic analysis (Braun & Clarke, 2022, p. 55). We generated initial themes (phase 3), which were later refined several times, going back and forth between the original text and the codes (phase 4). Themes in the reflexive thematic analysis involve the identification of patterns around ideas or concepts related to the research question (Braun & Clarke, 2022); they were led by the idea that they should tell the story, illustrate diversity and richness, and ultimately answer the research question. The research question was reformulated in parallel with the development of the themes. The first author prepared suggestions for themes for discussion among the authors, a discussion that did not aim to seek consensus but to gain richer and more nuanced insights into the material (Braun & Clarke, 2022, p. 55). Preliminary themes were presented and discussed in a reading circle on thematic analysis, and later in a group of experienced researchers in health educational research. The responses from both groups were discussed by the authors and considered in the refinement of the themes. The final themes were checked to ensure their uniqueness and boundaries (phase 5) and all themes contributed to the overall analysis. When writing the report (phase 6), citations were selected and translated from Norwegian to English by the first author. NVivo (NVivo, 2020), Excel, and MindManager (MindManager, 2019) were used as technical tools in the analysis process.

### Trustworthiness

Reflexivity in thematic analysis means being aware of your subjectivity and how it interacts with your research (Braun & Clarke, 2022). Reflexivity describes an active researcher making choices based on values, situated practice and in relation to methods and theory. This means that our analysis is always influenced by theoretical assumptions, not in an atheoretical vacuum. Reflexivity is an ongoing process of reflection during the whole process, never complete or finished. Furthermore, interpretation of the data happens through all phases, as the data the subjectivity of the researchers and the choices we make before and during data collection and all phases of analyses. We have sought credibility through describing the design of the study in detail, and the analytical process in a transparent manner. Dependability is taken care of through a stable research team during the whole study and concentrated time for data collection. The context and analysis are described in such detail, that readers can decide whether the findings are transferable to their setting.

### Ethical Considerations

All methods were performed in accordance with relevant guidelines and regulations, including institutional regulations and Guidelines for Research Ethics in the Social Sciences and the Humanities (NESH, 2021). The study was approved by the Norwegian Agency for Shared Services in Education and Research (ref. no.:45703) as required by the Personal Data Act (The Personal Data Act, 2018), on behalf of the institution hosting the study. The leaders at the institute approved conducting the interviews during the teachers’ working hours and on the university campus. Before the interviews, all participants were given written and oral information about the purpose of the study. Their participation was voluntary and confidential, and they had the right to withdraw at any time. All participants were given the opportunity to ask questions about the study, before providing written informed consent*.* The findings are presented in an anonymized manner to ensure confidentiality.

## Results

Twelve teachers were interviewed individually. Six had supervised students writing a literature review, and six had supervised students writing an empirical BT. Half of them had a master's degree and the other half had completed a PhD. Their experience of being BT supervisors varied between 5 and more than 15 years, except for one who had done this for the first time. They had supervised between three and 10 students each that term. There were 10 women and two men, aged around 40 years or older.

The analyses resulted in two generated themes, each with two subthemes, describing the patterns the teachers emphasized as important aspects of their supervision of nursing students writing their BT (Table 2). The themes of the pedagogical approach to the supervision and the advantages of the BT to the nursing profession are constructed by semantic and latent outcomes, respectively. The following section will present the results as an analytic narrative.

**Table 2.** Themes, Subthemes and Characteristics.

Themes	Subthemes	Characteristics
The pedagogical approach to the supervision	Individual approach: The importance of trust, confidence, and support	Meeting the students’ individual needs for support. Following the students’ ambitions and motivation. Trust and confidence in supervision meetings.
	Stimulating approach: Urging development through challenge and critical thinking	Challenging the students to develop and make progress. Discussion partners, encourage reflection and finding their own answers. Practicing critical thinking.
The advantages of the BT to the nursing profession	The professional nurse: Developing competence for clinical nursing practice	Clinical relevance is distinctly related to nursing. Deep knowledge of a theme of specific interest. Speaking up for their profession, communicating nursing.
	The academic nurse: Developing nursing as an academic profession	Critically thinking nurses, familiar with research methods, stimulating evidence-based practice. Assuring nursing as an academic profession, comparable with other professions.

*Note*. BT = bachelor’s thesis.

### The Pedagogical Approach to the Supervision

The pattern in this theme was the description of the pedagogical approach on which the teachers based their supervision. It revealed that the teachers were scaffolding the supervision with trust and individual orientation and filling it with challenge and the urge to develop.

#### Individual Approach: the Importance of Trust, Confidence, and Support

The teachers all highlighted the importance of a good relationship with each student, a relationship that should be based on the individual student's knowledge, ambitions, and motivations. The teacher's role was to find each student's level and adjust their supervision to the individual. Furthermore, the teachers emphasized building trust and confidence in the students and creating a good atmosphere for supervising. They supported the students through the use of acknowledgement and non-verbal communication. A common focus was to address the supervision of the student's interests and drive, to build their motivation and self-efficacy.

I try not to have a negative tone of voice but always start with something positive because there is always something good to say. (T6)

Some students struggle with low self-efficacy while others have high skills and are self-driven, so they require different supervision strategies. Therefore, we have to meet the students at their level and give them what they need as individuals. (T11)

#### Stimulating Approach: Urging Development Through Challenge and Critical Thinking

The teachers tried not to give the students answers but rather wanted them to find their own answers by thinking on their own. They asked questions in response to the student's questions and countered their arguments, recognizing that knowledge is made in the dialogue between teacher and student. They further motivated the students to be critical and creative about their writing process through dialogue and discussions. Reflection was seen as key to increasing the students’ understanding of their product. The teachers also pushed the students on time and workload and encouraged steady progression in the students’ work. Nevertheless, they were conscious not to be too harsh since the students were novices and inexperienced in writing assignments as large as the BT.

Learning might be a chaotic process, but I think it is important for them to learn how to articulate their own problem because then they might find a way to answer the problem themselves. (T9)

I am not afraid to challenge them and discuss with them: it's my experience that it makes them think and find their way out of the problem. Yes, it is about asking them good questions, but I can’t challenge them too much—most of them are pretty young and inexperienced. (T1)

### The Advantages of the BT to the Nursing Profession

The pattern of the second theme was how the teachers focused on the advantages of the BT beyond the learning outcomes related to the course. Where the learning outcomes mostly related to the individual student's knowledge, the teachers also focused further on what this would mean for the nursing profession, both in the clinic and in academia.

#### The Professional Nurse: Developing Competence for Clinical Nursing Practice

The nursing perspective on the theme for the BT was highlighted as important. It should be well-defined inside the nursing profession. Furthermore, the learning outcomes from the BT had to be clinically relevant. The BT was seen as an exceptional opportunity to dig deep into a specific field of interest, gain vulnerable insight into nursing practice, and discover and reflect on significant values in clinical practice. The BT enabled students to bridge the theory into a clinical setting, supporting practical competence and increasing clinical understanding. It collected all threads from education up till now. The teachers’ aim was for the students to have courage and confidence in their professional conversations, to talk about knowledge and research, to articulate the nursing profession, and to make their voices heard. They wanted the students to become communicating nurses, speaking up for their profession, both together with other nurses and in interprofessional discussions.

I encourage them to show why this theme or research question is important, and why is it important for nurses to know about this, how it contributes to a wider context. (T2)

If it doesn’t have any clinical relevance and you don’t learn anything practical from it, then I don’t find it of value … it's not worth anything if you can’t use it in the clinic. (T3)

Out there in the real world, you need to be confident in your decisions, to have the courage to raise your voice in interprofessional teams, or to argue against the doctor. I think it's a very important quality as a nurse, to communicate the nursing profession with a confident voice. (T4)

#### The Academic Nurse: Developing Nursing as an Academic Profession

Through the BT, the teachers saw an opportunity to supervise students to become nurses with academic capacity, to further ensure nursing as an academic profession. They wanted the students to learn how to produce an academic text of quality, to be confident with the research process, and to experience the joy of discovering or creating new knowledge. Furthermore, they wanted the students to become critically thinking nurses, being curious and asking questions, but also being humble in their own knowledge and understanding its limitations. The teachers aspired to develop nurses that contributed to knowledge-based nursing in practice. They also expressed the importance of comparability with other educational courses in terms of the requirements for the BT and the students’ preparedness for further education at the master's level.

I think that research and nursing belong together—not all the students are becoming researchers, but we must teach it to the students, so they understand our profession. I think teaching them research methods is important to help them become critically thinking nurses. (T10)

Developing knowledge and attitude toward knowledge is important because it never stops: everything continues during your career, I think, so that is why I think it is so important. (T8)

I hope they see the joy of exploring and being in the process, discovering new knowledge and working in a structured way, because I think the process is as important as the product. (T9)

## Discussion

The teachers in this study emphasized two important aspects regarding their supervision of nursing students writing a BT: their pedagogical approach to the supervision and the advantages of the BT to the nursing profession.

The teachers’ two pedagogical approaches to supervision may be labeled as interactional and transactional roles. The transactional role may be equivalent to the subtheme “stimulating approach,” where they describe taking a stimulating approach with a challenging attitude, critical thinking and striving for progression (Ädel et al., 2023). They encourage the students to have autonomy and independence in their process and emphasize the dissemination of knowledge. The institutional role is equivalent to the subtheme “individual approach”, which describes the teachers building an individual relationship with the students, relying on trust and confidence (Ädel et al., 2023). Both roles considerably echo the results of other studies (Agricola et al., 2018; Reguant et al., 2018; Roberts & Seaman, 2018).

The findings from this study support the argument that it is not sufficient to choose just one role or one direction in supervision. Thus, the quality of the supervision is dependent on the teachers’ ability to merge elements from different roles in supervision to be able to cover all needs of the students, depending on, for example, how independently the students work and where in the process they are (Ädel et al., 2023; Todd et al., 2006). The different roles need different strategies and confirm the complexity of supervision (Agricola et al., 2021), something which requires teachers who are both responsive and flexible and have high pedagogical skills. We see from other studies that balancing roles is reported as being a challenge for many supervisors (Agricola et al., 2018; Holmberg, 2006; Todd et al., 2006; Vehviläinen & Löfström, 2016), but in our study, the teachers do not express this. The teachers in this study present the supportive role as a safe foundation for being challenging, something that grounds the situation. When the teachers need to give direction and sometimes demand progression and independence, they can rely on the supportive relationship, built on trust and confidence, as a safe basis to tell the students what they may not want to hear and to adjust their feedback to the student's maturity and level of experience. This study may exemplify how interactional and transactional roles, even if they sometimes appear conflicting, are equally important and mutually dependent in the supervision of nursing students on their BT (Ädel et al., 2023).

The other theme found in this study was how the teachers expressed the advantages of BT for the nursing profession. A particular finding from this study is the teachers’ distinct focus on the clinical relevance of the BT, well defined inside the nursing profession, as described in the subtheme “the professional nurse.” The learning outcomes should be relevant and coherent with students’ future practice. This is interesting together with the results from another study which found that it is difficult for nurse academics to “let go” of their clinical past as they move into higher education settings (Barrow & Xu, 2021). This may be a reason for the distinct focus on being supportive and urging confidence and trust in the relationship, which are important qualities in nursing practice. The teachers act as supervisors the same way they want the students to act as nurses. They are demonstrating important aspects of clinical nursing, mirroring an identity for the students to learn from and incorporate into their own nursing practice (Baldwin et al., 2017). The teachers’ focus on clinical relevance in this study may confirm that the core identity of academic nurses is enacted through clinical practice (Barrow & Xu, 2021). The teachers’ focus on clinical relevance is consistent with an emphasis on the fitness to practice as a product of an educational process, almost more than on the pedagogical approach (Mackintosh-Franklin, 2016).

The teachers in this study also wanted to educate an academic nurse, able to contribute to the development of nursing as an academic profession, as described in the last subtheme. They wanted to provide the students with knowledge and skills to participate in research projects and utilize research knowledge, making them able to continue to develop their personal and professional knowledge. They wanted them to practice evidence-based nursing and to learn critical thinking. Critical thinking is a thread between the two main themes of this study. In training the students to think critically, the supervisors are forming critically thinking nurses capable of contributing to the nursing profession. When the teachers act as role models using critical thinking as a pedagogical tool, they stimulate the students to think and encourage them to come up with an answer themselves (Agricola et al., 2021; Borglin & Fagerström, 2012), and they arrange a safe place for the students to practice. The BT also scaffolds a nursing context for the students, which is essential for the development of critical thinking skills (Jefferies et al., 2018). The students need the nursing context, the clinical relevance, and the deep nursing knowledge; the BT is providing to develop their critical thinking adequately. The findings of this study confirm that the BT is a great means for nursing students to practice critical thinking (Borglin & Fagerström, 2012), and supervising the BT mediates the impact the teachers have on developing critical thinking nurses for the future.

The two themes of the professional versus the academic nurse show that the teachers find both themes to be important advantages for the nursing profession, which is the same dualism that they are working within, their two identities. Clinical and academic identity go together, the former a foundation for the latter, while research work may be seen as an extension of nursing practice (Barrow & Xu, 2021). Furthermore, academic nursing teachers and nurses in the clinic have a shared goal: to improve the lives of patients (Mitchell, 2018). Working with knowledge through the BT, through supervision or in clinical practice, should reflect that.

## Strengths and Limitations

In this study, the interviews were conducted by a PhD student at the faculty, which might have affected the interview in different ways. Some teachers might feel uncomfortable sharing their thoughts about their own work performance with a distant colleague, while others would rather discuss these themes with an acquaintance than with a stranger. Nevertheless, they all expressed a great willingness to contribute their experience, all voluntarily participating in the study. Using individual interviews also made it possible to dig deeper into the theme and to stop and take time to elaborate on parts of the interview, resulting in rich material (Polit & Beck, 2017). Even if qualitative research is not automatically generalized to other situations, the developed knowledge in this study may be useful for other teachers supervising nursing students or other professions at an undergraduate level.

## Implication for Practice

The findings of this study have shown the benefits for nursing students and the nursing profession when the teachers combine their clinical and academic identities. The nursing education must encourage the teachers to maintain their clinical identity, alongside developing their academic competence. Further, the findings from this study are an important contribution to future attempts to develop guidelines, role definitions, or other supporting materials for supervising undergraduate nursing students on their BT.

## Conclusion

This study has shown that what the teachers emphasize as important regarding their supervision of nursing students writing their BT is based on both their pedagogical approaches to the supervision and how they want the BT to advantage the nursing profession. When supervising, they combine interactional and transactional roles, and clinical and academic identities, with critical thinking as a thread through the findings, modeling the practice of critical thinking to form critically thinking nurses. The findings demonstrate that supervising nursing students on their BTs is not just a technical issue for the teachers but goes beyond: it is grounded in their nurse identity and their aspirations for the profession, which is not described elsewhere.
